# Multi-strain probiotic intervention in adults with depressive and anxiety symptoms: A 12-Week pilot study

**DOI:** 10.1038/s41398-026-04334-6

**Published:** 2026-08-06

**Authors:** Yaniv S. Ovadia, Tamer Okal, Dov Gefel, Amit Shama, Pnina Nir, Milena Pitashny, Daniela Amital

**Affiliations:** 1 Research Authority, Barzilai University Medical Center, Ashkelon, 7830604 Israel; 2 Robert H Smith Institute of Plant Sciences and Genetics, Faculty of Agriculture, The Hebrew University of Jerusalem, Rehovot, 7610001 Israel; 3 Division of Psychiatry, Barzilai University Medical Center, Ashkelon, 7830604 Israel; 4 Division of Infectious Diseases, Barzilai Medical Center, Ashkelon, 7830604 Israel; 5 Microbiology Laboratory, Hillel Yaffe Medical Center, Hadera, 3842401 Israel; 6 Faculty of Health Sciences, Ben Gurion University of the Negev, Beer Sheva, 8410501 Israel

**Keywords:** Depression, Scientific community

## Abstract

Depressive and anxiety symptoms prevalent in major depressive disorder (MDD), that affects millions globally, with many patients showing incomplete response to standard treatments. This open-label pilot study evaluated the effects of a 12-week multi-strain probiotic supplementation on depressive and anxiety symptoms in 30 adults with depressive and anxiety symptoms (e.g., MDD, adjustment disorder, anxiety disorders). Participants received a daily capsule for eight weeks containing 11 bacterial strains (9.8 billion CFU), including *Bifidobacterium animalis* ssp. *Lactis Lafti B94* (5 billion CFU) and *Lactobacillus rhamnosus* RO-11 (1.9 billion CFU). Outcomes were assessed using the Hamilton Depression Rating Scale (HDRS), Hamilton Anxiety Rating Scale (HAM-A), Generalized Anxiety Disorder 7-item scale (GAD-7), and Clinical Global Impression (CGI) scale at baseline, 4, 8, and 12 weeks following treatment. The intervention was well-tolerated, with minor gastrointestinal side effects in 10% of participants. Significant reductions were observed in HDRS (22.47 ± 6.95 to 8.83 ± 5.32, *p* < 0.01), HAM-A (32.27 ± 11.18 to 13.09 ± 10.49, *p* < 0.01), GAD-7 (15.33 ± 4.21 to 6.87 ± 5.37, *p* < 0.01), and CGI (4.53 ± 0.94 to 3.00 ± 1.65, *p* < 0.01) scores. Of note, females, participants with tertiary education, and married individuals showed greater improvements. The substantial reductions in these scores suggest probiotics may contribute in the reduction of depressive and anxiety symptoms, potentially via the microbiota-gut-brain axis, supporting further investigation in larger, controlled trials.

**Figure:**
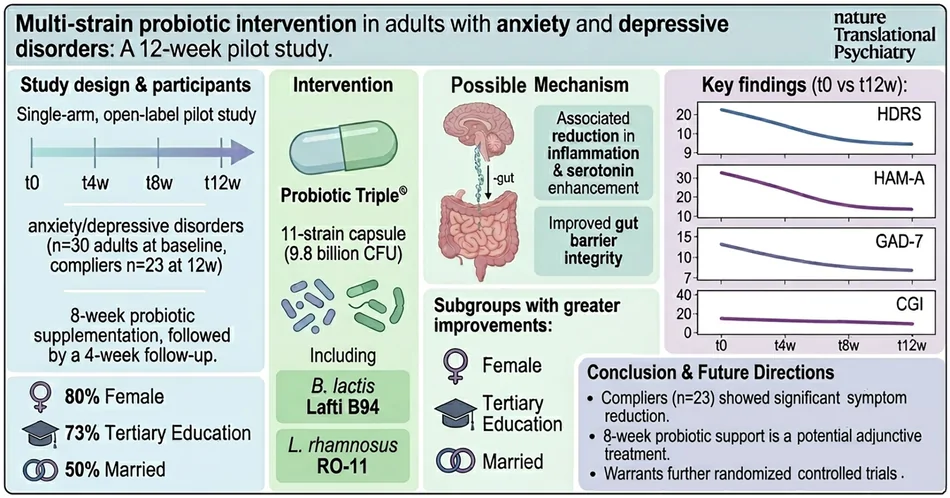

## Introduction

Depressive and anxiety symptoms are predominant and complicating factors in major depressive disorder (MDD), that affect over 280 million people globally, contributing to significant disability [1]. Current treatments, including antidepressants and psychotherapy, are effective for some but leave many with persistent symptoms [2]. Symptoms include low mood or loss of interest, often accompanied by guilt, hopelessness, and changes in appetite and sleep [3]. Also, pharmacological treatments for MDD focus on altering brain neurotransmitter activity, but have a delayed onset of action and might result in adverse side effects such as headaches, nausea, agitation, sedation, and sexual dysfunction [3]. Emerging evidence highlights the role of the microbiota-gut-brain axis in mental health, with gut dysbiosis implicated in MDD pathophysiology [4]. Probiotics, which have previously been shown to modulate gut microbiota, have shown promise in reducing depressive symptoms. We assumed that such daily consumption to modulate gut microbiota, could potentially achieve this via the microbiota-gut-brain axis. Clinical trials’ review suggested that treatment with probiotics may improve symptoms associated with MDD by increasing serotonin availability and/or decreasing levels of inflammatory markers [5]. However, less is known about specific probiotic compositions for MDD, including optimal bacterial strains, combinations, dosages, and treatment durations.

Current scientific literature addresses possible links between multistrain probiotics and MDD, mostly Bifidobacterium and Lactobacillus species [6, 7]. The most rigorously studied multistrain probiotic interventions in MDD have used daily doses of eight billion Colony Forming Units (CFU), but the specific strains and their ratios have varied and are not detailed to the level of a specific composition [4, 8]. Multi-strain formulations such as those including Bifidobacterium and Lactobacillus species were suggested to target diverse microbial pathways [9]. We hypothesized that daily consumption of a multistrain probiotic at doses greater than eight billion CFU may amplify anti-inflammatory and neuroregulatory benefits.

This pilot study aimed to evaluate the feasibility, safety, and preliminary efficacy of an eight-week multi-strain probiotic supplementation in adults with depressive and anxiety symptoms. We hypothesized that this intervention can reduce depressive and anxiety symptoms that were assessed by validated clinical scales, potentially via modulation of the microbiota-gut-brain axis.

## Methods

### Study design and setting

This single-arm, open-label pilot study took place over 12 weeks (total research period February 2024 to March 2025), with assessments at baseline (t0), 4 weeks (t4w), 8 weeks (t8w), and 12 weeks (t12w) at the outpatient clinic of the Division of Psychiatry, Barzilai University Medical Center, Ashkelon (BUMCA), Israel. The assessment included Hamilton Depression Rating Scale (HDRS), Hamilton Anxiety Rating Scale (HAM-A), Generalized Anxiety Disorder 7-item scale (GAD-7), and Clinical Global Impression (CGI) scores. All procedures were approved by the institutional review board and adhered to the Declaration of Helsinki. The study protocol number 0112-23-BRZ was approved by the BUMCA ethical committee (approvals dated February 11 2024). All participants signed informed consent after the research protocol was explained to them in detail.

### Participants

Thirty adults aged 18–65 years attending BUMCA’s Division of Psychiatry functioning outpatient clinic with depressive and anxiety symptoms [including diagnosis of MDD, adjustment disorder, anxiety disorder, persistent depressive disorder (PDD), or anxiety and depression disorder], confirmed by DSM-5 criteria, were recruited. Exclusion criteria included severe suicidal ideation, psychosis, substance use disorders (except volunteers using cannabis for medical purposes), chronic gastrointestinal conditions and significant medical comorbidities requiring acute intervention (full details are provided in Supplementary list 1). Participant characteristics are summarized in Table 1.

**Table 1:** Characteristics of the studied population at t0.

Parameter	Result
**Age (y)** mean ± SD	38 ± 12
**Gender** n(%)	
Female	24 (80%)
Male	6 (20%)
**Tertiary education** n(%)^***T***^	
None	8 (26.7%)
Tertiary education	22 (73.3%)
**Family status** n(%)	
Divorced	8 (26.67%)
Married	15 (50%)
Single	7 (23.33%)
**Medical background** n(%)	
Asthma	2 (6.7%)
Endometriosis	1 (3.3%)
Systemic lupus erythematosus	1 (3.3%)
Myopia (nearsightedness)	1 (3.3%)
Obesity	1 (3.3%)
Pre-diabetes	1 (3.3%)
None	23 (76.6%)
**Diagnosis** n(%)	
Adjustment disorder	19 (63.3%)
Anxiety and depression disorder	4 (13.3%)
Anxiety disorder	3 (10%)
PDD	2 (6.67%)
Major depression disorder	2 (6.67%)
**Current Cannabis use** n(%)^*M*^	3 (10%)
**Current drugs abuse** n(%)	0 (0%)
**Benzodiazepine use** n(%)	5 (16.67%)
**Antidepressant use** n(%)	9 (30.0%)
**medication name** n(%)	
Burpropion (Wellbutrin)	1 (3.33%)
Escitalopram (Cipralex, Esto)	6 (20.00%)
Mirtazapine (Miro)	2 (6.67%)
Sertraline (Lustral, Serenada)	1 (3.33%)
None	20 (66.67%)
**None Psychiatric medication use** n(%)	
Birth control pills	3 (10%)
Esomeprazole (Nexium)	2 (6.67%)
None	23 (83.33%)

^T^Tertiary education: ‘None’ = completion of primary/secondary education only; ‘Tertiary’ = university or equivalent post-secondary education.

^M^For medical purpose.

*t0*, time at baseline; *SD* standard deviation, *n* number, *%* percentage*/* proportion, *PDD* persistent depressive disorder.

### Intervention

Participants received a daily probiotic supplement (Probiotic Triple®), purchased by the research staff directly from the producer (Altman LTD, Or Yehuda, Israel). Capsules contained 11 bacterial strains from the *Lactobacillus* and *Bifidobacterium* genera (total 9.8 billion CFU per capsule):

- *Bifidobacterium animalis* ssp. *Lactis* Lafti B94: 5 billion CFU
- *Lactobacillus rhamnosus* RO-11: 1.9 billion CFU
- *Lactobacillus helveticus* RO-52: 1.35 billion CFU
- *Lactobacillus helveticus* Lafti L10: 0.5 billion CFU
- *Bifidobacterium longum* RO-175: 0.3 billion CFU
- *Bifidobacterium breve* RO-70: 0.2 billion CFU
- *Lactobacillus paracasei* RO-215: 0.2 billion CFU
- *Lactobacillus rhamnosus* GG: 0.2 billion CFU
- *Bifidobacterium infantis* RO-33: 0.15 billion CFU
- *Lactobacillus plantarum* RO-1012: 0.1 billion CFU
- *Lactococcus lactis* ssp. *Lactis* RO-1058: 0.1 billion CFU

Participants were advised to swallow a single capsule once daily with water for eight weeks, preferably with a meal (see details in Table 2). Each participant received at each visit an amount of 30 capsules for 30 days at t0 and t4w. Compliance was calculated based on self-reported consumption and returned capsules as follows:

$$C=\frac{{SC}\left(n\right)-{RC}\left(n\right)* }{28}$$

*RC(n)≤2 considered 0 for this calculation, as two spare capsules (above the 28 needed for intervention for 4 weeks each time) were supplied at t0 and t4.

Where: C, compliance; SC(n), supplied capsules number; RC(n), returned capsules number.

**Table 2:** Intervention follow-up and compliance scores.

Serial	t0 supplied (n)	t4 returned (n)	Compliance score	t4 supplied (n)	t8 returned (n)	t8 compliance score	Total compliance score	Drop timing	Self-reported side effects
1	30	0	1.00	30	0	1.00	1.00	-	None
2	30	0	1.00	30	1	1.00	1.00	-	None
3	30	0	1.00	30	0	1.00	1.00	-	None
4	30	1	1.00	30	4	0.93	0.96	-	None
5	30	0	1.00	30	2	1.00	1.00	-	None
6	30		0.00			0.00		Between t0 to t4	None
7	30	0	1.00	30	0	1.00	1.00	-	None
8	30	0	1.00	30	2	1.00	1.00	-	None
9	30	0	1.00	30	5	0.89	0.95	-	None
10	30	2	1.00	30	1	1.00	1.00	-	None
11	30	3	0.96	30	4	0.93	0.95	-	None
12	30	2	1.00	30	4	0.93	0.96	-	None
13	30	0	0.00	30	1	1.00	1.00	-	None
14	30	3	0.96	30	4	0.93	0.95	-	None
15	30	0	1.00	30	0	1.00	1.00	-	None
16	30	0	1.00	30	0	1.00	1.00	-	None
17	30	0	1.00	30	1	1.00	1.00	-	None
18	30	0	1.00	30	0	1.00	1.00	-	None
19	30	27	0.11	30		0.00	0.05	At t4	Abdominal pain
20	30	15	0.54	30		0.00	0.27	At t4	None
21	30	0	1.00	30	27	0.11	0.52	-	None
22	30	4	0.93	30	27	0.11	0.48	-	None
23	30	0	1.00	30	2	1.00	1.00	-	None
24	30	5	0.89	30		0.00	0.45	At t4	Bloating and diarrhea
25	30	3	0.96	30	4	0.93	0.95	-	None
26	30	0	1.00	30	0	1.00	1.00	-	None
27	30	1	1.00	30		0.00	0.52	At t4	None
28	30		0.00			0.00	0.00	Between t0 to t4	Bloating
29	30	0	1.00	30	4	0.93	0.96	-	None
30	30		0.00			0.00	0.00	Between t0 to t4	None

*t0* time at baseline, *t4w* time at four weeks of intervention, *t8w* time at eight weeks of intervention.

Any compliance score equal or below 0.52, as well as self-reported side effects due to intervention between t0 to t8 resulted in exclusion of participant’s data from the analysis regarding t12.

Concomitant medications (e.g., antidepressants, benzodiazepines, see detailed list at Table 1) were permitted if prescribed and taken routinely and constantly (without dose changes for at least 4 weeks prior to enrollment.

### Outcome measures

We used all validated assessment questionnaires as follows:

HDRS: A 17-item clinician-rated scale for depressive symptoms (range: 0–52) [10].

HAM-A: A 14-item clinician-rated scale for anxiety symptoms (range: 0–56) [11].

GAD-7: A self-reported measure of anxiety symptoms (range: 0–21) [12].

CGI: A clinician-rated scale for overall illness severity (range: 1–7) [13].

Assessments were conducted by blinded raters. Safety was monitored by research staff via participants’ self-reported side effects and through adverse event reporting assessments. Anxiety was assessed using both clinician-administered (HAM-A) and self-reported (GAD-7) tools to provide complementary perspectives, to enhance result robustness. Potential discrepancies were not adjusted, as trends were consistent across measures.

### Statistical analysis

Continuous variables were reported as means ± standard deviations, and categorical variables as frequencies and percentages. Repeated-measures ANOVA assessed changes in outcome measures over time, with post-hoc Tukey tests for pairwise comparisons. Subgroup analyses used ANOVA or Student’s t-tests, stratified by gender, education, family status, medical background, diagnosis, and medication use, when appropriate. To control for cannabis use, advanced models including cannabis use as a covariate were performed. Models included linear mixed-effects for each outcome scale (HDRS, HAM-A, GAD-7, CGI), incorporating current cannabis use (yes/no) and its interaction with Time point as fixed effects. Sensitivity analyses were also performed (Supplementary file S1) to assess the impact of cannabis users on results. P-values < 0.05 were considered significant.

### Software

JMP Pro software version 18 (SAS Institute, Cary, NC, USA) was used to calculate descriptive and selected advanced statistics (including Fixed Effects Tests and model diagnostics), after which R software (version 4.2.2; R Foundation for Statistical Computing, Vienna, Austria) was used for repeated-measures ANOVA, post-hoc tests, and correlation analyses.

## Results

### Participant characteristics and compliance

Total enrollment into the study included 30 participants (80% female, mean age 38.37 ± 11.81 years). The majority had tertiary education (73.3%) and were married (50%). Diagnoses included adjustment disorder (63.3%), anxiety and depression disorder (13.3%), anxiety disorder (10%), PDD (6.67%), and MDD (6.67%). Medical comorbidities were reported for 13.4% cases, with the rest reporting none (76.6%). Antidepressant use was reported in 30.0% cases (medication use distribution in Table 1). No drug abuse was reported. Detailed characteristics of the studied population at baseline are described in Table 1.

A total of 30 participants reported on following the intervention at t0, t4w and t8w. At t12w, seven participants reported non-compliance as shown in Table 2 and were excluded from advanced analysis (Tables 2 and 3).

**Table 3:** HDRS Scores by Subgroups.

Subgroup	t0	t12w	P-value*
**Participants** (n)	30	23	NS
**Gender**			
Female	24 ± 7	8 ± 5	<0.01
Male	19 ± 7	11 ± 6	0.08
**Tertiary Education**			
None	26 ± 7	13 ± 5	<0.01
Tertiary	21 ± 7	7 ± 5	<0.01
**Diagnosis**			
Adjustment disorder	24 ± 7	9 ± 6	<0.01
Anxiety and depression	22 ± 6	9 ± 3	0.01
Overall score	22 ± 7	9 ± 5	<0.01

^*^Each Pair Student’s t test.

± values are mean ± SD.

*HDRS* Hamilton Depression Rating Scale, t0 time at baseline, t12w time at 12weeks from intervention, SD standard deviation.

### Primary outcome: HDRS score

**Fig. 1:**
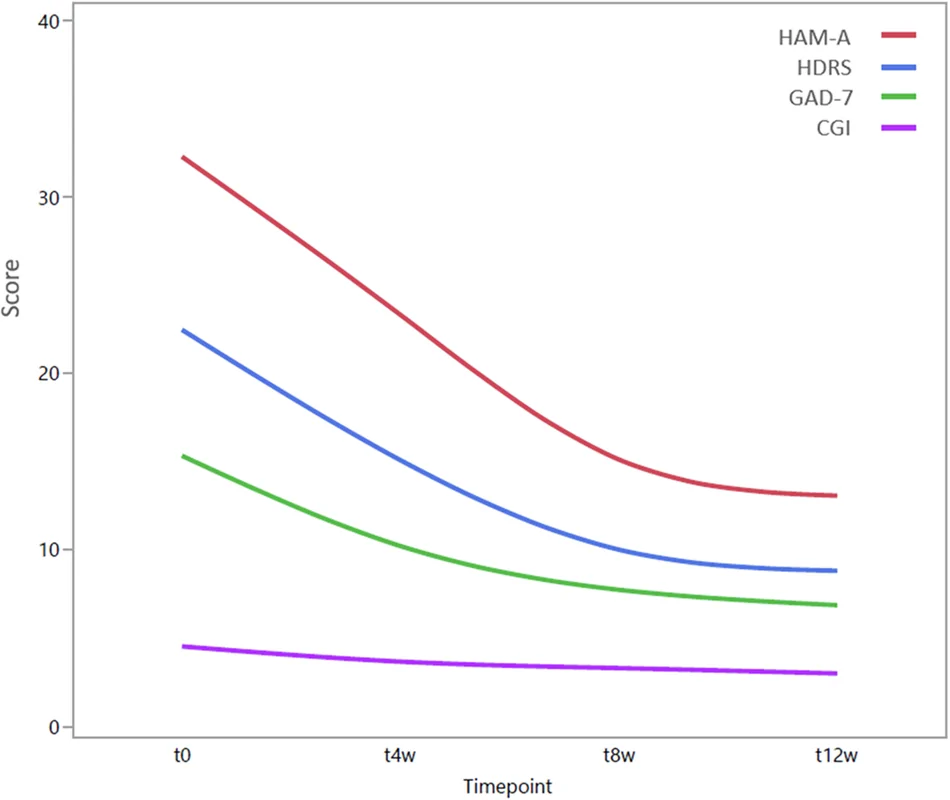
Line graph showing mean HDRS, HAM-A, GAD-7, and CGI scores over the study period. All scores declined significantly during intervention period, with HDRS showing the lowest P-value at t4w and t8w. Abbreviations: HAM-A, Hamilton Anxiety Rating Scale; HDRS, Hamilton Depression Rating Scale; GAD-7, Generalized Anxiety Disorder 7-item scale; GCI, Clinical Global Impression; t0, time at baseline; t4w, time at four weeks of intervention; t8w, time at eight weeks of intervention; t12w, time at 12 weeks from intervention.

**Fig. 2:**
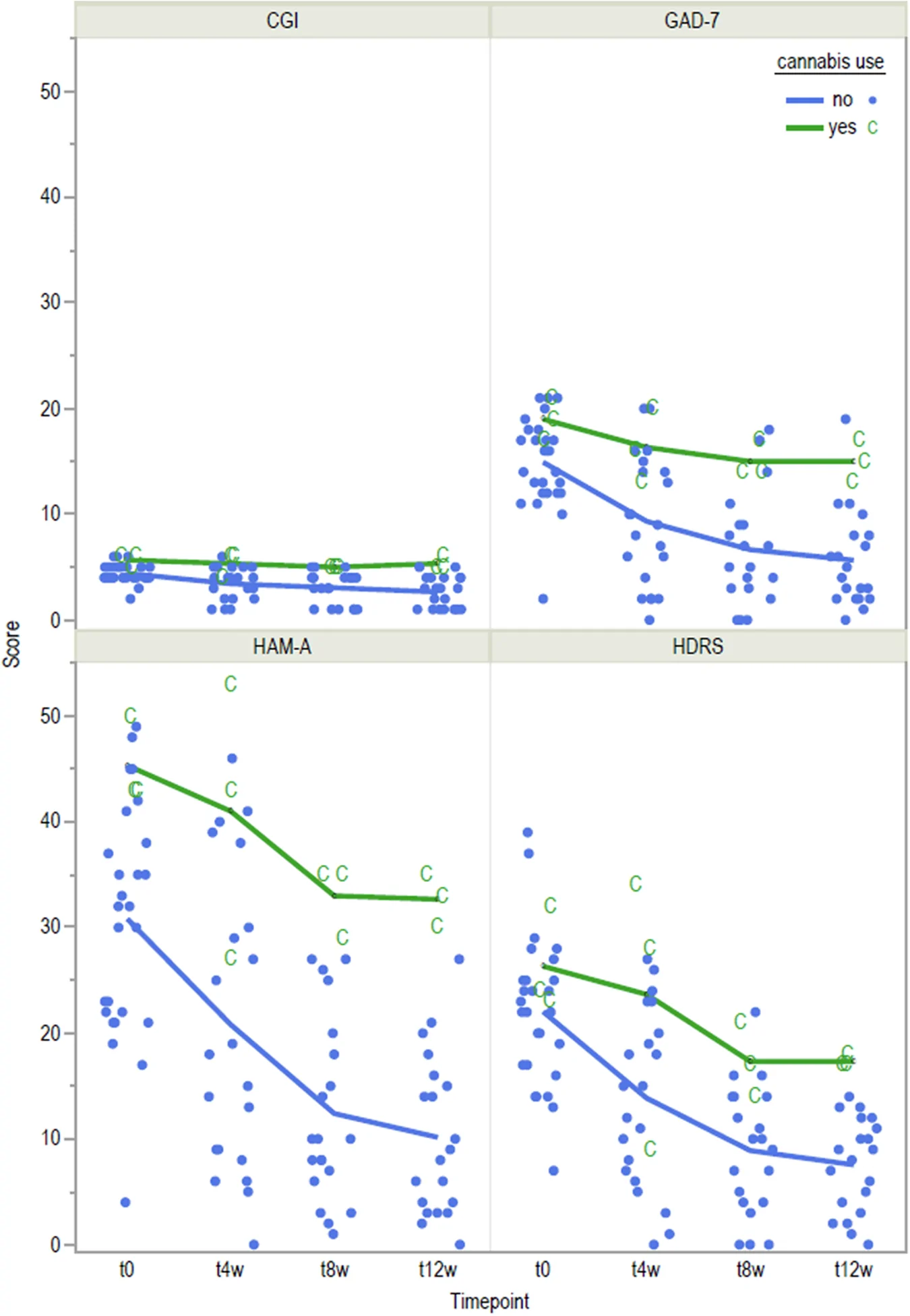
Scatter plots and mean pattern of CGI, GAD-7, HAM-A and HDRS scores over 12 weeks of intervention of none vs. cannabis users. Intersection points above each time point are means Abbreviations: : HAM-A, Hamilton Anxiety Rating Scale; HDRS, Hamilton Depression Rating Scale; GAD-7, Generalized Anxiety Disorder 7-item scale; GCI, Clinical Global Impression; t0, time at baseline; t4w, time at four weeks of intervention; t8w, time at eight weeks of intervention; t12w, time at 12 weeks from intervention.

The HDRS scores significantly decreased from baseline (t0) to the end of the follow up at 12 weeks (t12w). Significant reductions in these scores were also observed at 4 weeks (t4w) (15.08 ± 9.34, *p* < 0.01) and 8 weeks (t8w) (10.00 ± 6.47, *p* < 0.01). Figure 1 illustrates the decline in HDRS scores over the eight-week intervention period, showing a continuous reduction from baseline to t12w. Figure 2 further highlights that a reduction in means HDRS score was evident in both none vs. cannabis users. Subgroup analyses (Table 3) revealed significant improvements in females (*p* < 0.01), participants with tertiary education (*p* < 0.01), married individuals (*p* < 0.01), and those with adjustment disorder (*p* < 0.01). Also, Antidepressant users also showed improvement (*p* < 0.01) in HDRS score from t0w to t8w and t12w. In sub-group analysis only those on Cipralex (*n* = 6) showed such decrease (*p* < 0.01, Each Pair Student’s t test). No significant differences were found in HDRS changes between those on Cipralex vs. none antidepressant users throughout all timepoint of the study.

### Secondary outcomes

The HAM-A scores decreased significantly from 32.27 ± 11.18 at t0 to 13.09 ± 10.49 at t12w (*p* < 0.01). In line with the primary outcome, significant improvements were observed in females (*p* < 0.01), participants with tertiary education (*p* < 0.01), and those with adjustment disorder (*p* < 0.01). The GAD-7 scores also decreased significantly from 15.33 ± 4.21 at t0 to 6.87 ± 5.37 at t12w (*p* < 0.01). Significant reductions in GAD-7 scores were noted in females (*p* < 0.01) and married individuals (*p* < 0.01). The CGI scores showed a significant decrease from 4.53 ± 0.94 at t0 to 3.00 ± 1.65 at t12w (*p* < 0.01). Improvements in CGI scores were significant in females (*p* < 0.01) and participants with tertiary education (*p* < 0.01). Figure 1 visually represents the significant decline in HAM-A, GAD-7, and CGI scores throughout the intervention period.

### Cannabis use as a covariate

Detailed model outputs including cannabis use as a covariate are presented in Supplementary Tables S1–S4. The Timepoint × cannabis use interaction was non-significant in all scales (*P* > 0.17). The main effect of Timepoint remained statistically significant. Effect of no cannabis use (vs. cannabis use) showed a modest association with overall symptom severity (negative estimate) but did not alter the trajectory of change (when interaction with Timepoint, it was non-significant in each score, Tables S1-S4) table). Also, model diagnostics are shown in Supplementary Figures S1–S4. These figures present the actual versus conditional predicted scores from the linear mixed-effects models (with cannabis use included as a covariate). Re-running all mixed models after excluding the three cannabis use participants produced qualitatively and quantitatively identical conclusions (time effect remained significant at *P* < 0.01 for all scales; no material change in estimates or confidence intervals; detailed output available from the corresponding author upon request).

### Safety

No serious adverse events were reported. Gastrointestinal symptoms were documented for three participants (10%) as described in Table 2.

### Change over time

Change over time of CGI, GAD-7, HAM-A and HDRS(Fig. 2) showed parallel scores patterns of none vs. cannabis users from t0w to t12w. The mean delta HDRS score (t12w - t0) was −13.6 ± 5.2 (*p* < 0.01, Table 3).

## Discussion

### Principal findings

This pilot study demonstrates that an eight-week supplementation with “Probiotic Triple” significantly reduced depressive and anxiety symptoms in adults with depressive related symptoms. The multi-strain probiotic, rich in *Bifidobacterium* and *Lactobacillus* species, likely exerted effects via the microbiota-gut-brain axis, potentially by reducing inflammation, enhancing serotonin production, or improving gut barrier integrity [4, 14]. The significant reductions in HDRS, HAM-A, GAD-7, and CGI scores align with prior studies of probiotics in depression [5, 15]. Greater improvements in females, those with tertiary education, and married individuals may reflect higher compliance or psychosocial factors [16].

### The context of results

This pilot study’s findings, indicating significant reductions in depressive and anxiety symptoms following eight weeks of probiotic supplementation, are consistent with a growing body of evidence supporting the role of the microbiota-gut-brain axis in mental health. Multiple systematic reviews and meta-analyses have demonstrated that probiotics can reduce depressive symptoms, with several reporting significant standardized mean differences favouring probiotic interventions over placebo in patients with depression and anxiety disorders [9, 17–20]. For example, a randomized, double-blind, placebo-controlled trial reported clinical and metabolic improvements in patients with MDD following probiotic administration [8, 21]. The observed reductions in HDRS, HAM-A, GAD-7, and CGI scores in our study (Fig. 1) are in line with prior investigations, including recent randomized controlled trials and meta-analyses, which have shown that multi-strain probiotic supplementation - particularly those containing *Bifidobacterium* and *Lactobacillus* species - can significantly improve depressive and anxiety symptoms, possibly via mechanisms such as modulation of inflammation, enhancement of serotonin production, and improvement of gut barrier integrity [8, 17, 21].

### Research implications

The greater improvements observed by the aid of HDRS in females, those with tertiary education, and married individuals (Table 3) may reflect higher adherence or greater psychosocial support, though the literature has not yet fully elucidated the mechanisms underlying these subgroup differences [17, 20]. The heterogeneity of psychiatric diagnoses in this study, while a limitation, is also reflected in the meta-analytic literature, which suggests that probiotic interventions may have broad applicability across mood and anxiety disorders [17, 18, 20]. Also, the positive outcomes in antidepressant users, particularly those on escitalopram (20%, Table 1), are supported by recent randomized trials showing that probiotics can serve as a valuable adjunctive treatment to conventional pharmacotherapy, enhancing clinical response in patients with incomplete response to antidepressants [8] These findings highlight the need for larger, controlled trials to clarify clinical significance and optimal probiotic formulations.

### Clinical implications

This pilot study suggests that an eight-week multi-strain probiotic supplementation may serve as an effective adjunctive therapy for reducing depressive and anxiety symptoms in adults with MDD and related conditions, potentially via modulation of the microbiota-gut-brain axis. Hypothesized mechanisms include enhanced gut barrier integrity, reduced systemic inflammation, and increased serotonin production, as supported by prior research [4, 14, 21]. These findings have significant implications for clinicians, indicating that probiotics could be integrated into treatment plans, particularly for patients with incomplete responses to antidepressants, as seen in those on escitalopram [8]. The greater improvements observed in females, married individuals, and those with tertiary education suggest that psychosocial factors or adherence may influence outcomes, warranting tailored clinical approaches [8]. For policymakers, these results highlight the potential for cost-effective, low-risk interventions to address the global burden of MDD [1]. However, the open-label design and small sample size necessitate caution, as placebo effects or bias cannot be ruled out. If replicated in larger, randomized, double-blind, placebo-controlled trials, the present findings could provide a basis for integrating probiotic supplementation into conventional psychiatric practice, offering a potential adjunctive approach for the treatment of MDD. The absence of serious adverse events supports the safety of this intervention, encouraging further exploration as a complementary therapy.

### Strengths and limitations

This study utilized multiple validated outcome measures, including HDRS, HAM-A, GAD-7, and CGI, ensuring a comprehensive assessment of depressive and anxiety symptoms [18–21]. Additionally, it was comprised of relatively high participant compliance and a favorable safety profile, with no serious adverse events and only minor gastrointestinal side effects in 10% of participants (Table 2), probably highlighting the feasibility and low-risk nature of probiotic supplementation as an adjunctive therapy.

It is noteworthy that the detailed sensitivity analyses addressing cannabis (Supplementary Tables S1–S4), indicated that probiotic-associated symptom improvement over time was not moderated by cannabis use (Fig. 2). Also, model diagnostics (Actual by Conditional Predicted Plots, Supplementary Figures S1–S4) demonstrate high model fit across all scales when cannabis use was covaried. Under the below-mentioned limitations, these sensitive analyses can cautiously hint that the observed probiotic-associated improvements are not driven by the small subgroup using cannabis.

Limitations include the small sample size, lack of placebo control, and open-label design, which may introduce bias. Also, the heterogeneity of diagnoses and concomitant medications could confound results [17, 20]. An additional asymmetry exists in symptom assessment: while anxiety was evaluated with both clinician-rated (HAM-A) and self-reported (GAD-7) instruments, depression was assessed only with the clinician-rated HDRS. Moreover, the lack of detailed clinical history data, such as illness duration, current episode length, and full psychiatric treatment history, probably poses a further limitation [22]. This reasonable constraint could influence responsiveness and generalizability. Hence, randomized, double-blind, placebo-controlled trials with larger samples and microbiota profiling are required to confirm efficacy and clarify microbiome-brain axis mechanisms.

### Conclusions

An eight-week probiotic supplementation via a capsule containing 11 bacterial strains significantly reduced depressive and anxiety symptoms in 23 adults with depressive and anxiety symptoms. These findings support probiotics as a potential adjunctive treatment, for MDD or as a preferred psychotherapy strategy, meriting further research.

## Supplementary information

supplementary information

## Data Availability

Anonymized data are available upon request from the corresponding author.
