# Supplementary material for: Multi-strain probiotic intervention in adults with depressive and anxiety symptoms: A 12-Week pilot study
Source: Transl Psychiatry. 2026 Aug 6;16:497. doi: 10.1038/s41398-026-04334-6 (PMC13624256; doi:10.1038/s41398-026-04334-6)
Supplement: Supplementary file 1 [file 41398_2026_4334_MOESM1_ESM.pdf]

Supplementary Information

for

Multi-strain Probiotic Intervention in Adults with Depressive and Anxiety symptoms: A 12-Week Pilot Study

Supplementary List 1: exclusion* criteria grouped by categories

Psychiatric and neurological conditions: Severe suicidal ideation, psychosis, bipolar disorder, epilepsy, or uncontrolled seizures.

Substance use: History of drug or alcohol addiction in the past 6 months.

Gastrointestinal conditions: Any acute or chronic diagnosis that may affect microbial composition, (e.g. active bowel disease, current intestinal infection, including malignancies, IBD, IBS and positive *Helicobacter pylori* tests).

Medical comorbidities: Immunocompromised state, unstable severe illness (e.g., cancer, heart, kidney, lung, or coagulation disorders), or recent electroconvulsive therapy (within 1 year).

Recent medication or supplement use: Oral or intravenous antibiotics (within 4 weeks), any probiotic products or probiotic-enriched foods (within 2 weeks), or laxative use.

Allergies: Known or self-perceived hypersensitivity to milk, yeast, or soy.

Reproductive status: Pregnancy, lactation, or planned pregnancy during the study.

*Conditions were based on self-report and documentation within each volunteer's electronic medical file.

Abbreviations: IBD. Inflammatory bowel disease; IBS, Irritable bowel syndrome.

Supplementary Tables S1–S4: Full mixed-effects model results (cannabis covaried)

Table S1. CGI scores (n=30, 4 timepoints)

Term	Estimate	SE	DFDen	t Ratio	Prob > |t|	Lower CI	Upper CI
Intercept	4.376	0.382	25.6	11.44	<.0001*	3.589	5.162
current Cannabis use[no]	–0.958	0.382	25.6	–2.50	0.0190*	–1.745	–0.171
Timepoint[t0]	0.661	0.244	24.6	2.71	0.0122*	0.158	1.165
Timepoint[t4w]	–0.001	0.169	24.3	–0.00	0.9962	–0.350	0.349
Timepoint[t8w]	–0.314	0.154	27.1	–2.04	0.0517	–0.630	0.002
current Cannabis use[no] × Timepoint[t0]	0.328	0.244	24.6	1.34	0.1918	–0.176	0.832
current Cannabis use[no] × Timepoint[t4w]	–0.001	0.169	24.3	–0.00	0.9962	–0.350	0.349
current Cannabis use[no] × Timepoint[t8w]	0.020	0.154	27.1	0.13	0.8993	–0.296	0.336

<u>Fixed Effects Tests</u>
- current Cannabis use: F = 6.27, P = 0.0190*
- Timepoint: F = 3.20, P = 0.0386*
- Cannabis use × Timepoint: F = 1.73, P = 0.1841

Individual fixed-effect parameters were tested with t-tests; overall effects (e.g., Timepoint, Cannabis use) were tested with F-tests.

*Statistically significant (linear mixed-effects model, α=0.05)

Abbreviations: SE, standard error; DFDen = denominator degrees of freedom; CI, 95% confidence interval.

Table S2. HAM-A scores (n=30, 4 timepoints)

**Term**	**Estimate**	**SE**	**DFDen**	**t Ratio**	**Prob > |t|**	**Lower CI**	**Upper CI**
Intercept	28.468	2.456	30.7	11.59	<.0001*	23.458	33.479
current Cannabis use[no]	–9.532	2.456	30.7	–3.88	0.0005*	–14.542	–4.521
Timepoint[t0]	9.606	2.073	29.9	4.63	<.0001*	5.371	13.840
Timepoint[t4w]	2.551	2.223	24.9	1.15	0.2621	–2.029	7.132
Timepoint[t8w]	–5.433	1.402	38.0	–3.87	0.0004*	–8.271	–2.594
current Cannabis use[no] × Timepoint[t0]	2.272	2.073	29.9	1.10	0.2818	–1.963	6.507
current Cannabis use[no] × Timepoint[t4w]	–0.449	2.223	24.9	–0.20	0.8417	–5.029	4.132
current Cannabis use[no] × Timepoint[t8w]	–0.433	1.402	38.0	–0.31	0.7593	–3.271	2.406

Fixed Effects Tests

- current Cannabis use: F = 15.07, P = 0.0005*
- Timepoint: F = 16.34, P < 0.0001*
- Cannabis use × Timepoint: F = 0.82, P = 0.4944

Individual fixed-effect parameters were tested with t-tests; overall effects (e.g., Timepoint, Cannabis use) were tested with F-tests.

*Statistically significant (linear mixed-effects model, α=0.05)

Abbreviations: SE, standard error; DFDen = denominator degrees of freedom; CI, 95% confidence interval.

Table S3. GAD-7 scores (n=30, 4 timepoints)

Term	Estimate	Std Error	DFDen	t Ratio	Prob > |t|	Lower CI	Upper CI
Intercept	12.747	1.314	26.3	9.70	<.0001*	10.047	15.448
current Cannabis use[no]	–3.586	1.314	26.3	–2.73	0.0112*	–6.286	–0.886
Timepoint[t0]	4.216	1.109	27.9	3.80	0.0007*	1.943	6.488
Timepoint[t4w]	0.009	0.902	25.3	0.01	0.9926	–1.848	1.865
Timepoint[t8w]	–1.862	0.752	31.8	–2.48	0.0188*	–3.394	–0.330
current Cannabis use[no] × Timepoint[t0]	1.549	1.109	27.9	1.40	0.1737	–0.724	3.822
current Cannabis use[no] × Timepoint[t4w]	0.009	0.902	25.3	0.01	0.9926	–1.848	1.865
current Cannabis use[no] × Timepoint[t8w]	–0.529	0.752	31.8	–0.70	0.4870	–2.060	1.003

<u>Fixed Effects Tests</u>
- current Cannabis use: F = 7.44, P = 0.0112*
- Timepoint: F = 7.11, P = 0.0010*
- Cannabis use × Timepoint: F = 1.13, P = 0.3517

Individual fixed-effect parameters were tested with t-tests; overall effects (e.g., Timepoint, Cannabis use) were tested with F-tests.

*Statistically significant (linear mixed-effects model, α=0.05)

Abbreviations: SE, standard error; DFDen = denominator degrees of freedom; CI, 95% confidence interval.

Table S4. HDRS scores (n=30, 4 timepoints)

Term	Estimate	Std Error	DFDen	t Ratio	Prob > |t|	Lower CI	Upper CI
Intercept	17.142	1.488	39.6	11.52	<.0001*	14.133	20.150
current Cannabis use[no]	–4.025	1.488	39.6	–2.71	0.0100*	–7.033	–1.017
Timepoint[t0]	7.044	1.715	32.0	4.11	0.0003*	3.549	10.538
Timepoint[t4w]	1.589	1.695	24.2	0.94	0.3577	–1.907	5.085
Timepoint[t8w]	–3.979	1.047	44.1	–3.80	0.0004*	–6.088	–1.869
current Cannabis use[no] × Timepoint[t0]	1.877	1.715	32.0	1.09	0.2820	–1.617	5.371
current Cannabis use[no] × Timepoint[t4w]	–0.911	1.695	24.2	–0.54	0.5957	–4.407	2.585
current Cannabis use[no] × Timepoint[t8w]	–0.145	1.047	44.1	–0.14	0.8902	–2.255	1.964

<u>Fixed Effects Tests</u>
- current Cannabis use: F = 7.32, P = 0.0100*
- Timepoint: F = 12.10, P < 0.0001*
- Cannabis use × Timepoint: F = 0.64, P = 0.5948

Individual fixed-effect parameters were tested with t-tests; overall effects (e.g., Timepoint, Cannabis use) were tested with F-tests.

*Statistically significant (linear mixed-effects model, α=0.05)

Abbreviations: SE, standard error; DFDen = denominator degrees of freedom; CI, 95% confidence interval.

Supplementary Figures S1–S4: Model diagnostics (Actual by Conditional Predicted Plots)

Figure S1. CGI scores (Actual vs. Conditional Predicted)

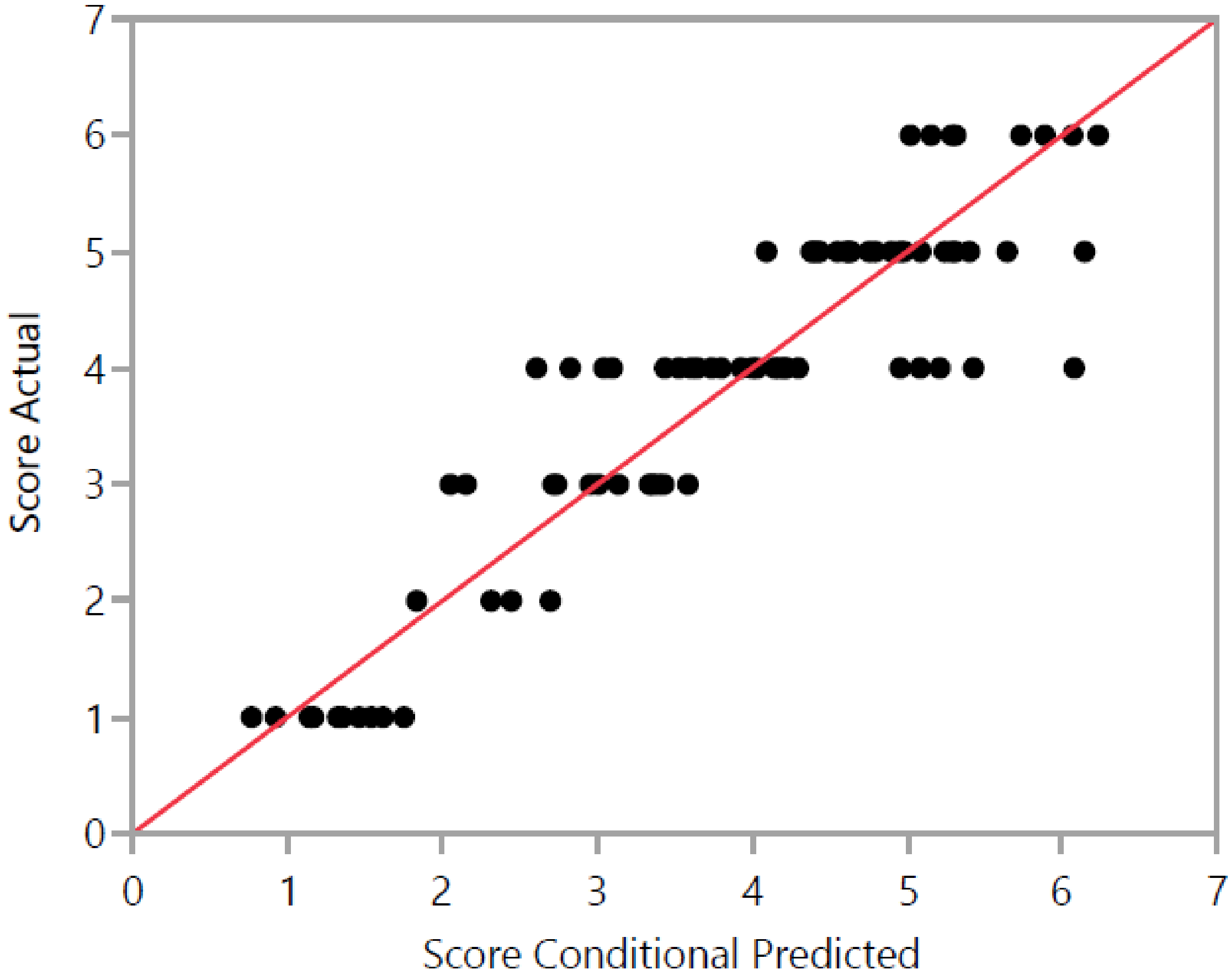

red line = fit

Figure S2. HAM-A scores (Actual vs. Conditional Predicted)

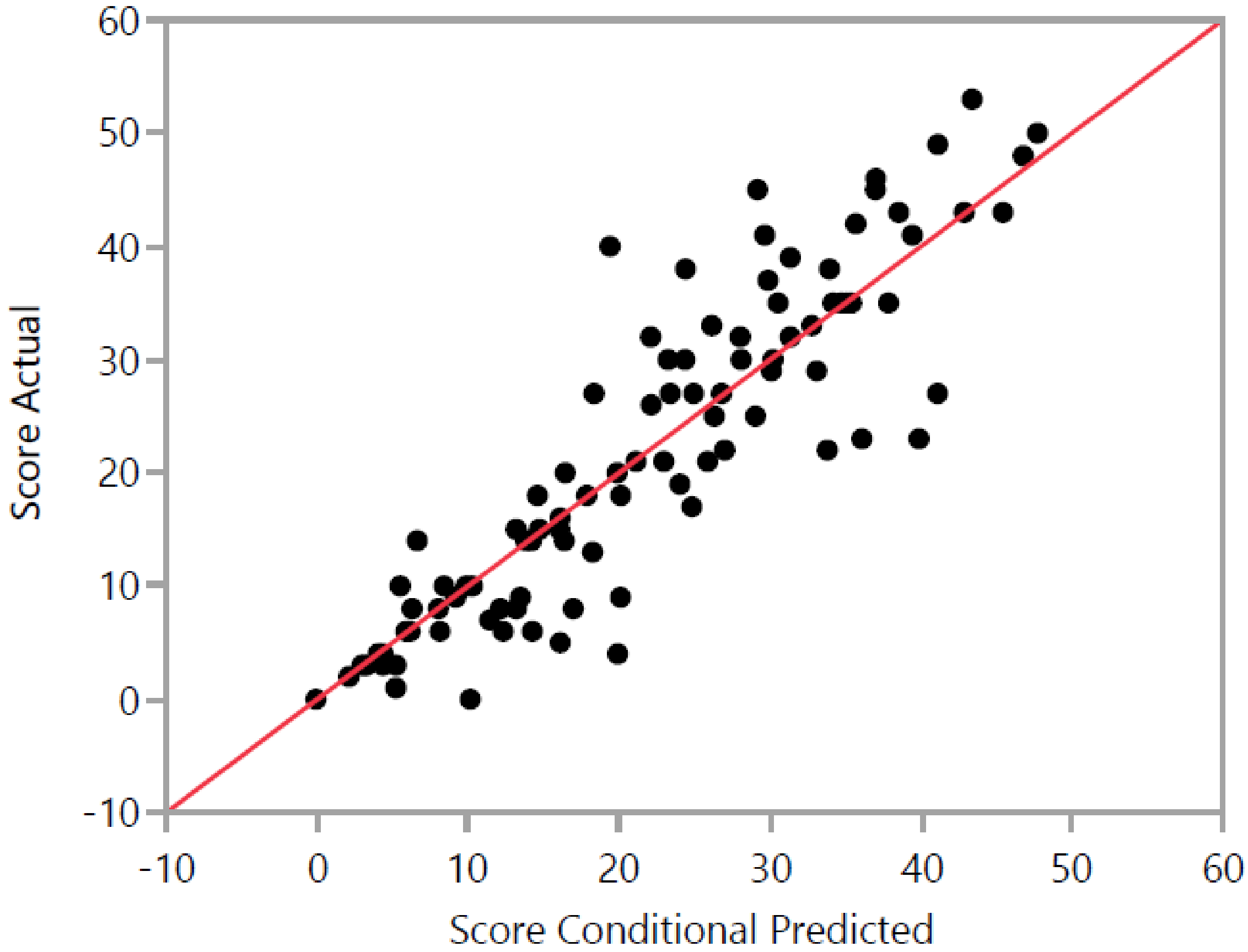

red line = fit

Figure S3. GAD-7 scores (Actual vs. Conditional Predicted)

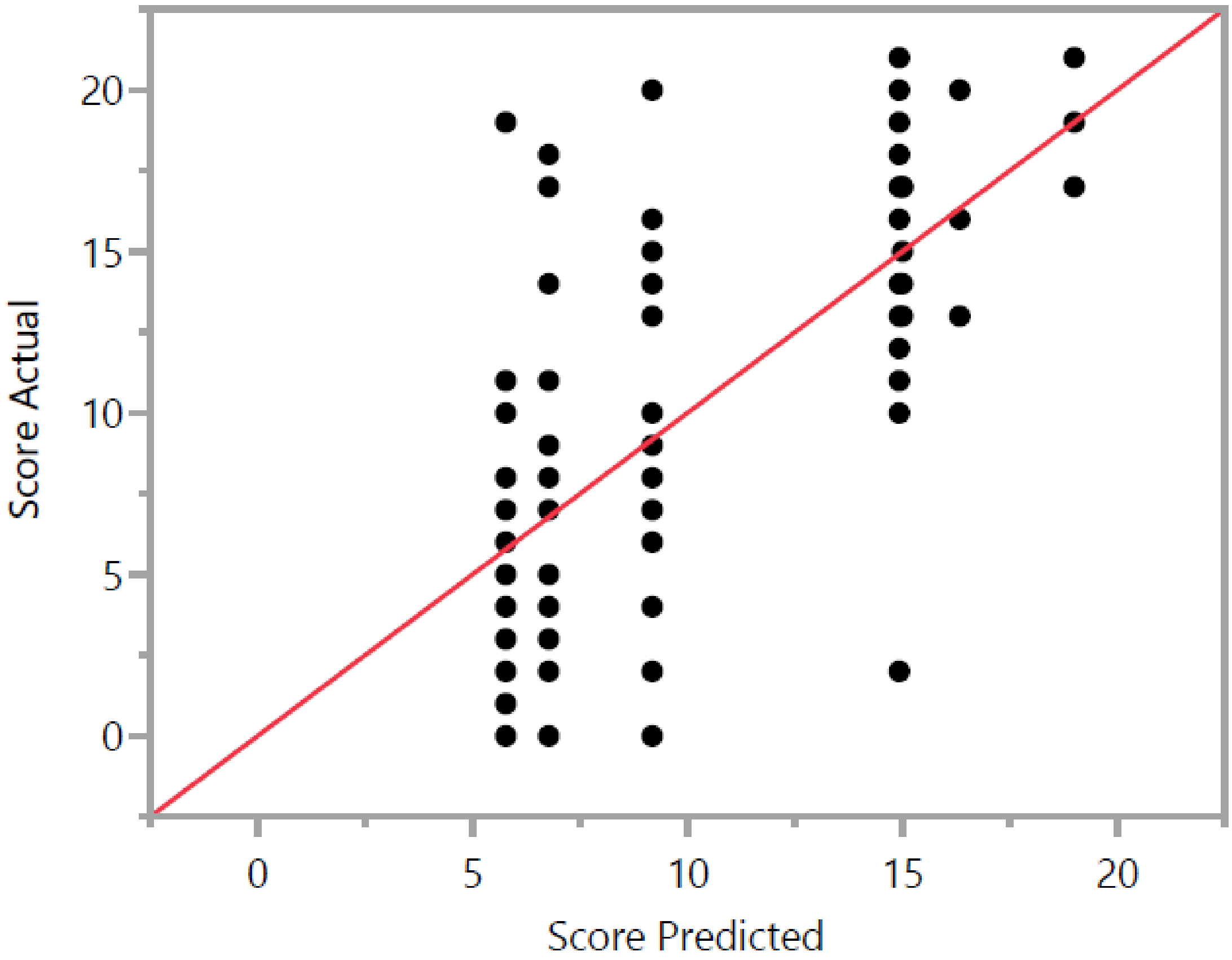

red line = fit

Figure S4. HDRS scores (Actual vs. Conditional Predicted)

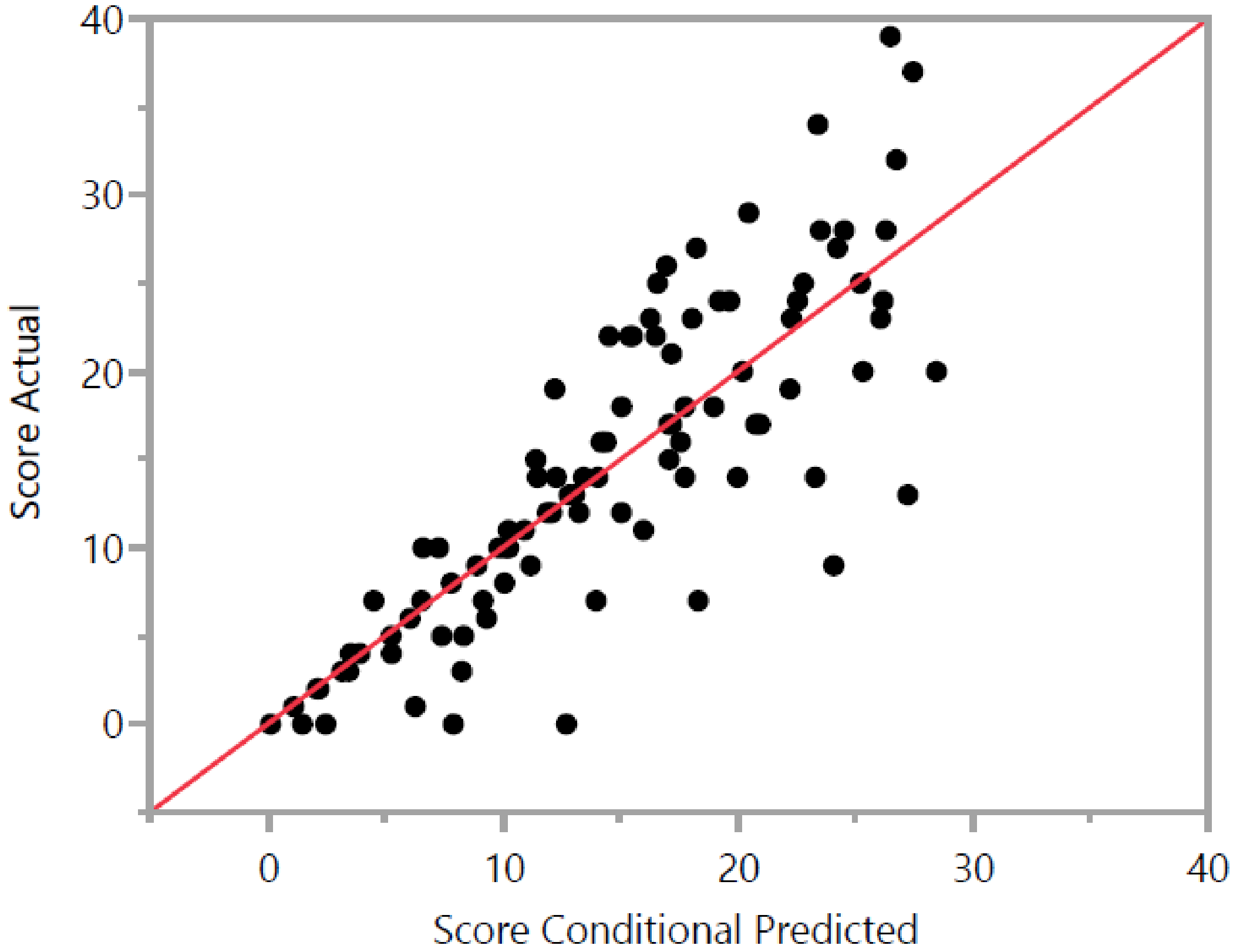

red line = fit
